# Phocine Distemper Outbreak, the Netherlands, 2002

**DOI:** 10.3201/eid1112.050596

**Published:** 2005-12

**Authors:** Jolianne M. Rijks, Marco W.G. Van de Bildt, Trine Jensen, Joost D.W. Philippa, Albert D.M.E. Osterhaus, Thijs Kuiken

**Affiliations:** * Dutch Wildlife Health Center, Rotterdam, the Netherlands; † Faculty of Veterinary Medicine, Utrecht, the Netherlands; ‡ Erasmus Medical Center, Rotterdam, the Netherlands; § Royal Veterinary and Agricultural University, Frederiksberg, Denmark

**Keywords:** Epidemiology, Phocine distemper virus, *Phoca vitulina*, the Netherlands, dispatch

## Abstract

During the 2002 phocine distemper epidemic, 2,284 seals, primarily harbor seals (*Phoca vitulina*), were found stranded along the Dutch coast. Stranding pattern varied with age, sex, state of decomposition, wind, and location. Cumulative proportion of deaths (54%) was comparable to that in the first reported epidemic in 1988.

Marine mammal morbilliviruses are among the most pathogenic infectious agents to emerge in wildlife. Phocine distemper virus (PDV) infection (*1**–**3*) was considered responsible for the deaths of ≈18,000 seals in Europe in the first recorded outbreak in 1988 (*4*), and of ≈22,000 seals in the second outbreak in 2002 (*5**,**6*). We examined the effect of different variables on the dynamics of the 2002 PDV epidemic in the Netherlands. This epidemic started 6 weeks after the first cases were noted on Anholt Island, Denmark (*5*). Subsequently, the disease spread east to Germany and Denmark, and west to Belgium, France, the United Kingdom, and Ireland (*6*). We also compared the epidemiologic characteristics of the 1988 and 2002 PDV epidemics in the Netherlands.

Seal strandings were reported to a central telephone service. Live stranded seals were rehabilitated or euthanized. Dead stranded seals were collected for necropsy during which species, sex, standard body length, and state of decomposition were determined. Seals were divided into age categories, based on sex and standard body length (*7*): male juveniles (age <1 yr; length <95 cm), subadults (1 yr < age <4 yr; 95 cm < length <140 cm), or adults (age >4 yr; length >140 cm); female juveniles (age <1 yr; length <90 cm), subadults (1 yr < age <3 yr; 90 cm < length <130 cm), or adults (age >3 yr; length >130 cm) (Table 1).Of 1,315 seals that underwent necropsy, complete data were obtained for 1,096 harbor seals (*Phoca vitulina*) (Table 1). These seals originated from the entire Dutch coast, except from the islands Rottumeroog and Rottumerplaat, where they were buried; from the island of Texel, where they were collected for a different study; and from the mainland coasts of North Holland and South Holland, where only a few seals were submitted for necropsy (Table 1, Figure 1A). Because seals on which a necropsy was performed represented 56%–73% of the stranded seals in the remaining locations (Table 1, Figure 1A) and had a similar-shaped epidemic curve to that of stranded seals (Figure A1), they were considered representative of stranded seals. The daily wind factor was calculated by multiplying average daily wind force at Den Helder, North Holland (obtained from the Royal Netherlands Meteorological Institute [KNMI]), with its coefficient. Coefficients were positive for winds north of the line west-southwest–east-northeast, negative for winds south of this line, and ranged from 0, when the wind direction was parallel to this line, to 4, when at right angles to it. To analyze the effect of spring tide, the number of strandings on the day of spring tide and the 2 subsequent days was compared to the number of strandings on other days. We used the χ^2^ test for categorical comparisons and linear trends, and Mann-Whitney U and Kruskal-Wallis tests for temporal scales, with pair-wise comparison for the variables that showed significant overall effect (SPSS for Windows, SPSS Inc., Chicago, IL, USA). For stranded seals with missing observations, age category, sex, and state of decomposition were imputed by using data on seals that underwent necropsy and had been stranded in the same location and on the same or closest weekly date.

**Table 1:** Age and sex distribution of harbor seals stranded during 2002 phocine distemper virus epidemic in the Netherlands

Stranding location	Coastline (km)*	Harbor seals							
		No. stranded	No. that underwent necropsy†	Juvenile‡		Subadult‡		Adult‡	
				M	F	M	F	M	F
Texel	57	281	–	–	–	–	–	–	–
Vlieland	41	303	198	25	35	86	123	11	23
Terschelling	67	338	190	42	36	92	134	17	17
Ameland	49	279	156	22	22	87	96	34	18
Schiermonnikoog	29	331	219	33	31	82	101	52	32
Rottumeroog and Rottumerplaat	14	172	–	–	–	–	–	–	–
Friesland mainland	93	217	159	8	21	57	66	27	38
Groningen mainland	80	194	138	18	34	28	50	24	40
North Holland	124	97	–	–	–	–	–	–	–
South Holland	36	18	–	–	–	–	–	–	–
Zealand	116	51	36						
Total	706	2,284	1,096	148	179	432	570	165	168

*Source: ref (*8*).

†These are harbor seals for which complete data were obtained. Seals from Texel, Rottumeroog and Rottumerplaat, North Holland, and South Holland were excluded because of the small proportions that underwent necropsy.

‡Age category and sex for stranded seals with missing observations were imputed by using those of seals that were stranded in the same location and on the same or closest weekly date.

**Figure 1:**
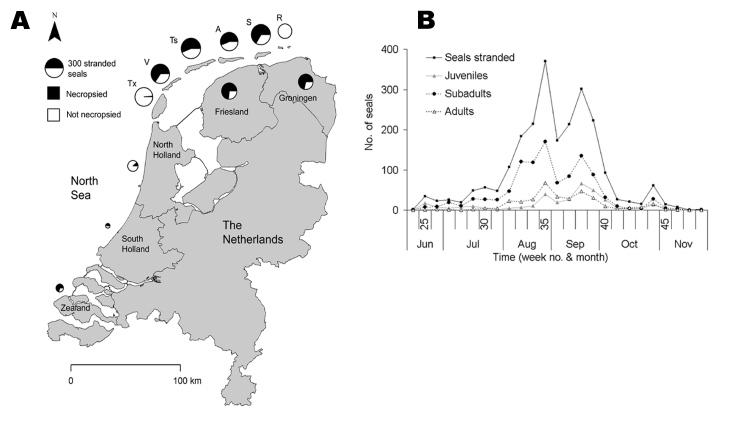
Spatial and temporal distribution of seal strandings in the Netherlands during the 2002 phocine distemper virus epidemic. A) Spatial distribution of seal strandings and proportion of seals necropsied at each location. The diameter of each pie chart corresponds to the number of seals stranded at a particular location. The names of the Wadden Sea islands have been abbreviated (Tx, Texel; V, Vlieland; Ts, Terschelling; A, Ameland; S, Schiermonnikoog; R, Rottumeroog and Rottumerplaat). B) Weekly stranding rate of all stranded harbor seals and effect of age category on weekly stranding rate.

Between June 16, 2002, when the Dutch index case was found on Vlieland, and the end of November 2002, when the stranding rate returned to preepidemic levels, 2,284 seals (2,154 dead, 130 live) were stranded along the Dutch coast (Figure 1B, Table 1). Almost all (2,279 of 2,284) were identified as harbor seals, and the remaining 5 as gray seals (*Halichoerus grypus*), despite recently increased gray seal numbers in the Netherlands and their likely exposure to PDV. This finding is consistent with experimental findings that PDV infection is more pathogenic for harbor seals than for gray seals (*9*). At gross necropsy, ≈80% of harbor seals had pulmonary consolidation consistent with PDV infection, while about 50% had either immunoglobulin M to morbillivirus by serology, morbillivirus-specific nucleic acid by reverse-transcription polymerase chain reaction (PCR), or both (unpub. data). This PCR fragment corresponded to that of PDV by phylogenetic analysis (*5*). Together these results confirm PDV infection as the primary cause of the epidemic. The rapid course of the epidemic, high cumulative proportion of deaths, and involvement of all age categories (Table 1) fit with a virgin soil epidemic and correspond with lack of preexisting specific immunity to PDV in most of the seals (*5**,**6*).

Age and sex affected temporal distribution of strandings. The median stranding date varied significantly among age categories (p<0.001). The date was significantly earlier for subadults than for juveniles and adults (p<0.05; Figure 1B). Subadults display considerably more social play than seals in other age categories, especially in early summer (*10*). In contrast, juveniles and their mothers are relatively more separated from other seals during the lactation period, are more sedentary, and have fewer new contacts (*11**,**12*). The median stranding date for males was significantly earlier than that for females in juveniles (p<0.001), subadults (p<0.001), and adults (p<0.001) (Figure A1 and A2). The average change in the number of individually identified seals hauled out between consecutive days is significantly higher for males than for females (*12*), and both subadult and adult males have the longest and most aggressive interactions with each other (*13*). These behavioral differences suggest that contact rates and intensity of contact with other seals, including seals with PDV infection, were higher for subadults at the start of the epidemic than for juveniles and adults, and higher for males than females, thus increasing the risk and severity of infection. Alternatively, the above patterns may be linked to age-related and sex-related differences in the effects of contaminants. The contaminant levels in the tissues of seals that died in the 1988 PDV epidemic were considered sufficiently high to cause immunosuppression and thus to increase the severity of the PDV outbreak (*14*). Pollutant levels in tissues of seals that died during the 2002 PDV epidemic have yet to be reported.

Age affected geographic distribution of strandings. The proportion of stranded seals of each age category varied significantly among Wadden Sea locations (p<0.001). The highest proportions of juveniles and adults stranded at mainland Groningen (Table 1), which includes Eemsmond, a core breeding area. The highest proportion of subadults stranded on Vlieland (Table 1) in the western part of the Dutch Wadden Sea, an area assumed to have an influx of migrating young seals (*15*). The number of seals stranded per kilometer of coastline varied significantly among locations for juveniles (p<0.001), subadults (p<0.001), and adults (p<0.001), with 2.2 to 3.1 more seals stranded per kilometer of coastline on Schiermonnikooog, an island in the eastern part of the Dutch Wadden Sea, than would be expected had the seals been evenly distributed per km coast (Table 1). This coincides with the summer distribution of harbor seals in the Dutch Wadden Sea, which is highly skewed toward the east (*15**,**16*). Within each age category, the proportion of males to females varied significantly among locations only for adults (p<0.001). Ameland had the highest proportion of adult males, and Vlieland the lowest (Table 1).

Location affected the temporal distribution of strandings. The median stranding date varied significantly among locations (p<0.001); that for Zealand (week 39) was significantly later than that for all Wadden Sea locations (weeks 35–37) (Figure A2). This is likely because seals in Zealand are fewer and more widely dispersed than in the Wadden Sea, so the chance of the virus spreading is lower.

Wind appeared to have a confounding effect on stranding rate: periods of southerly wind corresponded with decreased overall stranding rates, e.g., in weeks 33 and 36, and the opposite for northerly winds (Figure 2A). This is probably because dead seals floated in the top water layer, which shows parallel drift to surface winds. A similar effect of wind on strandings has been shown for seabirds (*17*). Spring tide did not affect stranding rate (p>0.05).

**Figure 2:**
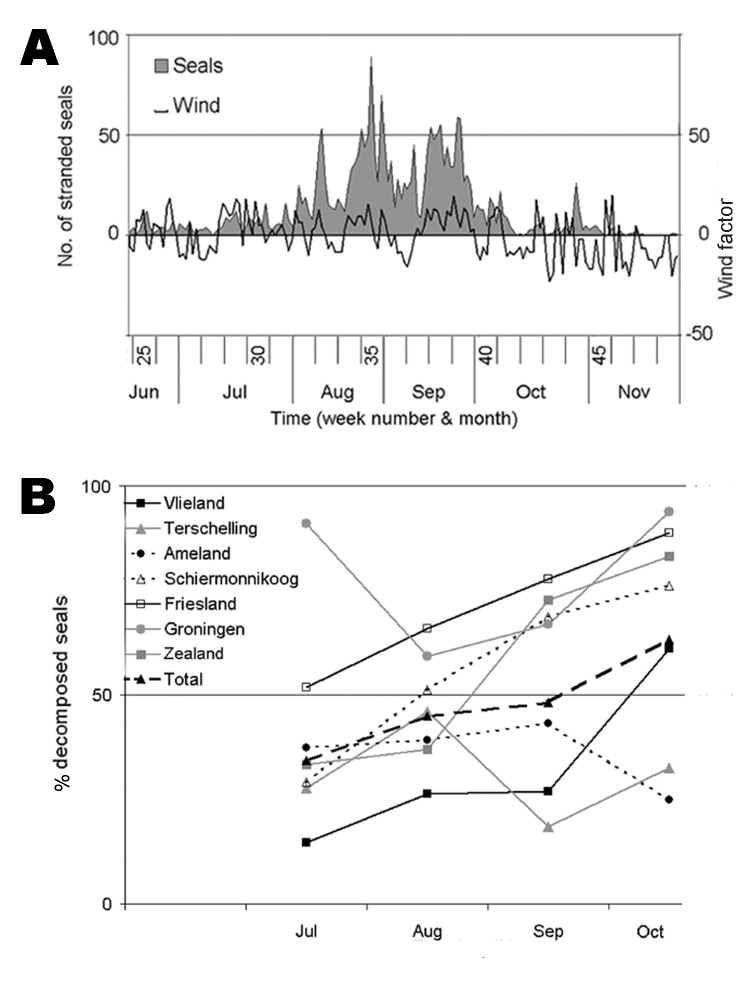
Effects of environmental variables on seal strandings in the Netherlands during the 2002 phocine distemper virus epidemic. A) Effect of wind direction and force on temporal distribution of stranded seals. Stranding rate of seals is expressed as number of seals reported per day. The wind factor is a function of wind force and wind direction. Negative wind factors correspond to southerly winds. B) Effect of state of decomposition on temporal distribution of stranded harbor seals, overall and per location. Percentages of decomposed seals are expressed per month.

State of decomposition (as a measure of length of time between death of a seal and its detection) also had a confounding effect on stranding rate. From July to October, the overall proportion of decomposed seals differed significantly among months (p<0.001) and increased significantly with time (p<0.001) (Figure 2B). This finding is probably because recovery of seal carcasses was not 100% so that, as the epidemic progressed, a higher proportion of stranded carcasses consisted of seals that had died before the previous shore survey. These findings show that over time stranding rate became a less accurate estimate of mortality rate, as observed in 1988 (*18*). The proportion of decomposed carcasses varied significantly by location (p<0.001) (Figure A3), with high proportions of decomposed carcasses on the mainland coasts of Friesland and Groningen and on Schiermonnikoog (Figure A4).

The timing of deaths and cumulative proportion of deaths of the 2002 PDV epidemic were similar to those characteristics in 1988 (Table 2). A difference, however, was that the index case was detected ≈1 month later in 2002 than in 1988. The similarity between estimated cumulative proportion of deaths in 1988 (53% of the population) and 2002 (54%) suggests that the pathogenicity of PDV for the harbor seal population has not changed noticeably. However, more detailed examination of the genetic composition of both the virus and the harbor seal is needed to exclude changes in the host-pathogen relationship.

**Table 2:** Comparison of overall characteristics of the 1988 and 2002 phocine distemper virus epidemics in the Netherlands

Variable	1988	2002
Date index case	May 22*	Jun 16
Date median case	Sep 4*	Sep 2
Central epoch (d)	115*	93
No. found stranded	417*	2,284
No. counted in preepidemic year	966†	3,595‡
No. counted in postepidemic year	535†	2,365‡
Average annual population growth in preepidemic years (%)	8§	19¶
Estimated cumulative proportion of deaths (%)#	53	54

*Source: ref (*4*).

†Source: Report "Compilatie van gegevens over zeehonden en zeehondenopvang in de Nederlandse Waddenzee" (available at http://www.waddenzee.nl/fileadmin/content/Dossiers/Natuur_en_Landschap/pdf/zeehondenplatform_compilatierapport.pdf).

‡Source: Wadden Sea Newsletters 2001-3 and 2003-2 (available at http://www.waddensea-secretariat.org).

§Source: ref (*19*).

¶Source: ref (*15*).

#Calculated as follows: [(number of live seals counted in preepidemic year + average annual growth in preepidemic years) – (number of live seals counted in postepidemic year – average annual growth in preepidemic years)]/(number of live seals counted in preepidemic year + average annual population growth in preepidemic years).

## Figures and Tables

**Figure A1:**
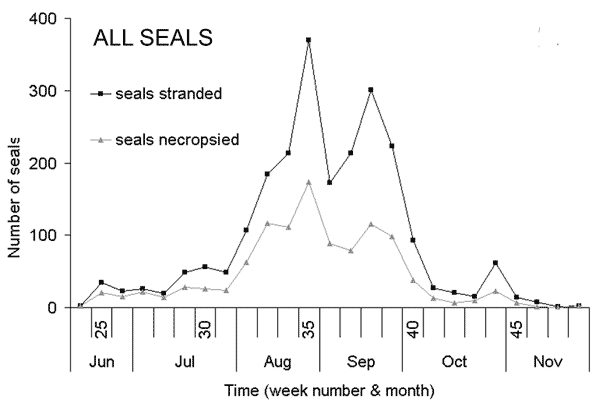
Weekly stranding rate of all recorded stranded seals compared to that of seals that underwent necropsy.

**Figure A2:**
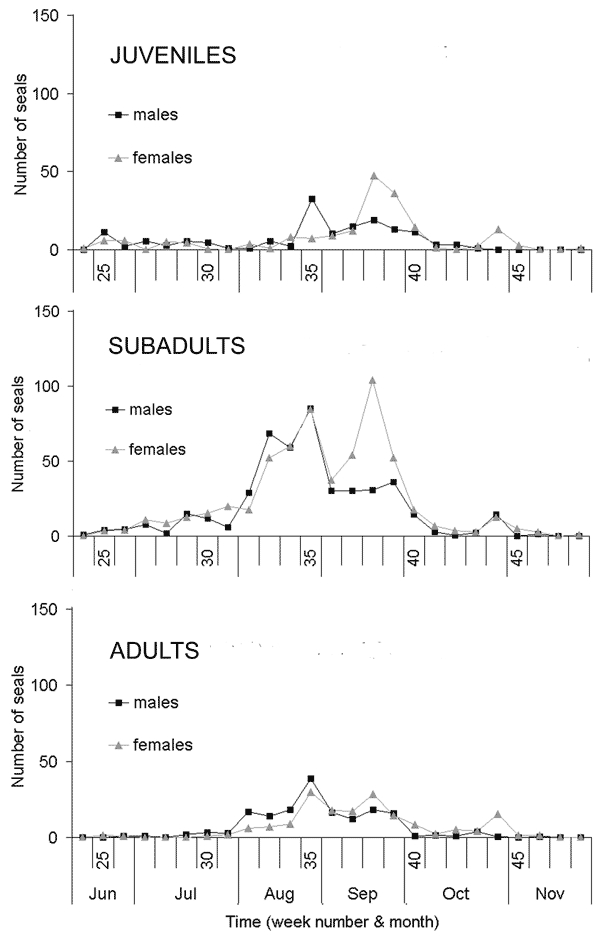
Weekly stranding rate of stranded harbor seals in each age category, by sex.

**Figure A3:**
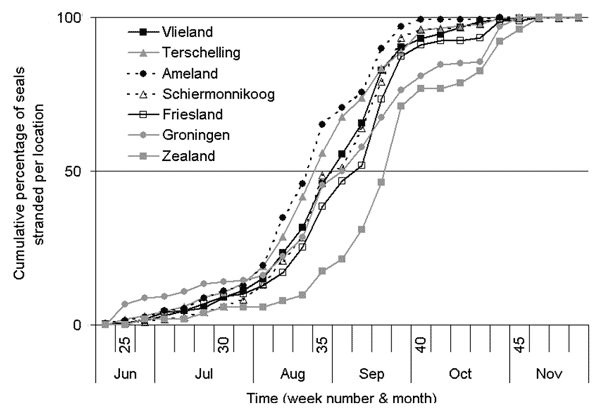
Effect of location on temporal distribution of stranded harbor seals. Strandings per location are expressed as a relative cumulative frequency curve. The 50% value of each curve corresponds with the median stranding date for a particular location. Note that strandings at Zealand occur ≈1 month later than at Wadden Sea locations.

**Figure A4:**
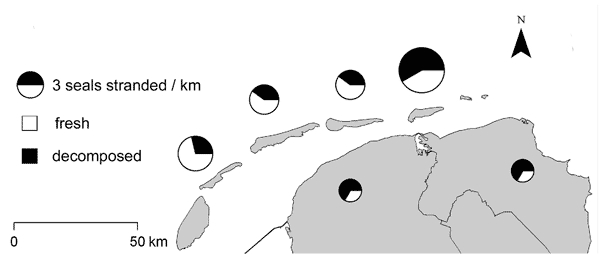
Effect of state of decomposition of stranded harbor seals. The diameter of each pie chart corresponds to the number of seals stranded per km of coastline at a particular location.
